# Biochemical composition of salivary stones in relation to stone- and patient-related factors

**DOI:** 10.4317/medoral.22533

**Published:** 2018-09-28

**Authors:** Saskia Kraaij, Henk S. Brand, Erik H. van der Meij, Jan-Gam de Visscher

**Affiliations:** 1 Department of Oral and Maxillofacial Surgery / Oral Pathology VU University Medical Center and Academic Centre of Dentistry Amsterdam (ACTA), Amsterdam, the Netherlands; 2 Department of Oral Biochemistry, Academic Centre for Dentistry Amsterdam (ACTA), Amsterdam, the Netherlands; 3 Department of Oral and Maxillofacial Surgery, Medical Centre Leeuwarden, Leeuwarden, the Netherlands

## Abstract

**Background:**

Salivary stones are calcified structures most often found in the main duct of the submandibular or parotid salivary gland. They contain of a core surrounded by laminated layers of organic and inorganic material.

**Material and Methods:**

Submandibular and parotid sialoliths (n=155) were collected at the department of Oral and Maxillofacial surgery of a general hospital between February 1982 and September 2012. The weight of the sialoliths was determined and the consistency was subjectively classified. Subsequently, the biochemical composition of the stones was determined by wet chemical methods or FT-IR spectrometry. Age and gender of the patients were retrieved from their medical records. Data were statistically analyzed using Fisher’s exact tests.

**Results:**

Sialoliths are mainly composed of inorganic material. Carbonate apatite was identified in 99% of the stones, phosphate in 88%, calcium in 87%, magnesium in 68%, struvite in 44%, oxalate in 38% and carbonate in 35%. Solid salivary stones contain more frequently struvite than stones with a soft consistency (*p*=0.05). Larger stones (>100mg) contain more frequently carbonate (*p*=0.05). Stones from older patients (≥38years) showed an almost significant trend towards more frequent presence of phosphate (*p*=0.083).

**Conclusions:**

The biochemical composition of submandibular and parotid sialoliths is related to stone-related factors, probably to age but not to the gender of the patient.

** Key words:**Salivary stone, sialolith, biochemical composition, FT-IR spectrometry.

## Introduction

Salivary stones are calcified structures most often found in the main duct of the submandibular or parotid salivary gland, which may cause mechanical obstruction associated with stasis of the saliva in the duct and gland. Associated symptoms are (mealtime related) recurrent swelling, pain and sometimes as a result, inflammation of the gland. Salivary stones, also called sialoliths, are most frequently located in the submandibular duct and salivary gland (72-95%) and less frequently in the parotid duct and gland (4-28%) (1). The sublingual and minor salivary glands are rarely affected. The mean annual incidence of hospital admission for patients with symptomatic sialolithiasis in the United Kingdom va-ries between 27.5 and 59 per million population per annum (2). Sialolithiasis is most common in patients in the fourth and fifth decade of life and is equally distributed between men and women. 

The etiopathogenesis of salivary stones is not completely understood. There are three main theories: agglomeration of sialomicroliths, calcification of a mucus plug and an altered biochemical composition of saliva (1). Su and co-workers (3) found that the saliva of patients with salivary stones is supersaturated with calcium and unsatiated with citrate, phytate and magnesium. It is assumed that salivary stasis or a decreased salivary flow contributes to the precipitation of calcium.

Submandibular and parotid salivary stones have similar structures. They consist of an amorphous, mineralized core surrounded by concentric laminated layers of organic and inorganic material. A very small percentage of sialoliths, submandibular as well as parotid, only consist of a core. The diameter of the nucleus varies between 0.5 and 1.5mm and is usually homogeneous but may contain substructures. These substructures refer to the proposed pathogenesis of sialoliths by agglomeration of sialomicroliths. These differences in structure and build-up may cause differences in colour and hardness of salivary stones (4,5).

The composition of salivary stones can be analyzed with different techniques: wet chemical techniques, X-ray powder diffraction and/or infrared spectroscopy (FT-IR) (4,6). X-ray diffraction and infrared spectroscopy offer the best identification of components, are fast and reproducible whereby infrared spectroscopy is becoming the gold standard (7,8). The infrared spectrum originates from the vibrational motion of the molecules. The vibrational frequencies are a kind of fingerprint of the compounds. This property is used for the characterization of organic and inorganic compounds present in calculi.

Knowledge of the biochemical composition of salivary stones is essential for understanding their etiology. Therefore, the aim of the present study was to investigate whether the inorganic biochemical composition of salivary stones is related to stone-related factors (size, consistency) and / or patient-related factors (age and gender of patient).

## Material and Methods

- Patients and stones

In the period from February 1982 to June 1996 the department of Oral and Maxillofacial Surgery of the Medical Centre Leeuwarden obtained 67 salivary stones from 67 patients (36 men and 28 women, 3 gender unknown) (group 1). The mean age of these patients was 37 years (age range 4-79). All these stones were from ducts of submandibular salivary glands.Between March 1997 and September 2012 another series of 88 salivary stones from 87 patients (45 men and 41 women, 1 gender unknown) was collected (group 2). The mean age of this patient group was 47 years (age range 8-87). Nine of the salivary stones from this series were from the paro-tid gland (10%) and 69 from the submandibular salivary gland (78%), the origin of ten stones was not registered. 36 percent of the salivary stones in group 2 were from the left and 52 percent from the right side.

All stones were removed through sialendoscopy, a transoral approach or by surgical removal of the affected gland. After removal, the stones were washed with distilled water and stored in plastic jars.

During the course of this study, all guidelines and protocols of the Declaration of Helsinki were followed (9).

- Stone analysis

Salivary stones collected in the first period (group 1) were quantitative analyzed using wet chemistry methods as described by Larsson *et al.* (1984) (10). Wet chemical analysis is based on the quantification of ions and organic components, from which the quantitative composition of the components can be calculated. Ions and organic material such as calcium, magnesium, ammonium, oxalate, phosphate, carbonate, urate and cystine can be detected (11). Stones obtained in the second period (group 2) were subjectively classified by an Oral and Maxillofacial surgeon as solid or soft. Subsequently, they were analyzed by Fourier Transform Infrared spectrometry (FT-IR) (mid infrared region 4000-400 cm-1) using the KBr disk technique (12,18). The size of a peak in the spectrum corresponds exactly with the quantity of a specific compound. Qualitative estimations of the presence of whewelliet, wheddeliet, carbonate apatite, struvite, brushite, cystine, ammonium urate and proteins were obtained.

- Statistical analysis

Statistical analysis was performed using IBM SPSS Statistics for Windows version 24.0 (IBM Inc, Armonk, NY), using Fisher’s Exact 2-sided tests and Spearman’s rank order coefficient. *P* values < 0.05 were considered statistically significant.

## Results

**Figure 1:**
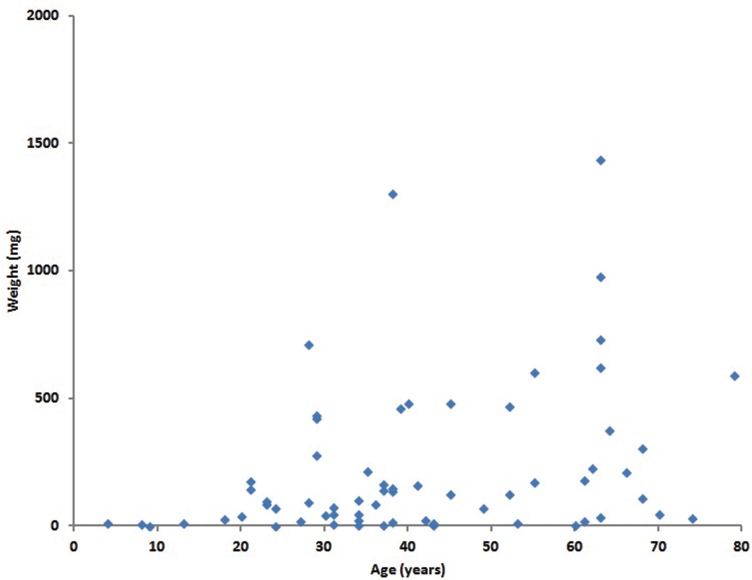
Relation between weight of the salivary stone and the age of the patient. Spearman rank order coefficient, (r = 0.382, *p* = 0.002) (n=66).

The salivary stones collected during the first period had an average weight of 418mg (s.d. 1278). The weight of the salivary stone correlated significantly with the age of the patient (r = 0.382, *p* = 0.002) (Figure 1). The components in these salivary stones, identified by wet chemical analysis, are presented in  Table 1. Most of the stones contained phosphate (88.4%), calcium (87.0%) and magnesium (68.1%). Carbonate and oxalate were present in approximately one third of the stones. Ammonium, cystine and urate were rarely detected (<3%). The biochemical composition of the salivary stones collected during the second period was determined using FT-IR. Nearly all these salivary stones contained carbonate apatite (98.9%) and in approximately a half of the stones struvite was present (43.7%). Wheddeliet (9.2%), whewelliet (3.5%), brushite (5.6%), ammonium urate (1.2%) and proteins (1.2%) were rarely identified. Cystine was not detected in any of the salivary stones.

**Table 1:**
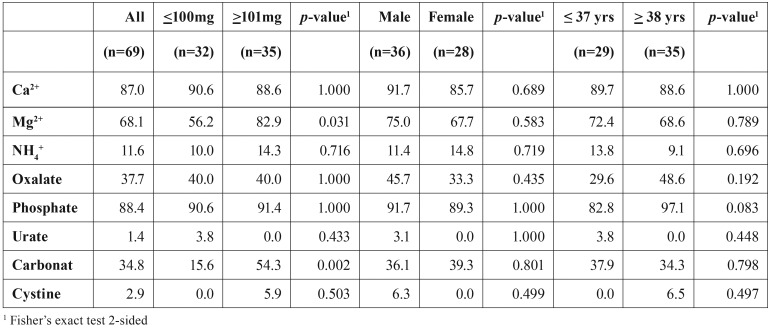
Biochemical analysis of salivary stones, stratified according to size of the stone and gender and age of the patient. The number in the table indicate the percentage of the salivary stones that contain the specific component.

The salivary stones from group 1 were dichotomized, based on the median weight of 100 mg, into two groups: ‘low weight’ (≤100mg, n=32) and ‘high weight’ (≥ 101mg, n=35). Stones from the ‘high weight’ group contained more frequently magnesium and carbonate than stones from the ‘low weight’ group. Percenta-ges oxalate and phosphate were almost equal for both groups ( Table 1).

Based on the median age, the patients from the first period were also stratified in two groups: ‘young’ (≤ 37 years, n=29) and ‘old’ (≥ 38 years, n=35). Stones from ‘old’ patients contained more phosphate than stones from the ‘young’ group. This difference almost reached statistical significance (*p*=0.083) ( Table 1). The stones collected during the second period were also stratified according to the mean age (47 years) into ‘young’ (≤ 47 years, n=44) and ‘old’ (≥ 48 years, n=43) No age-related differences were observed in the biochemical parameters determined by FT-IR ( Table 2).

**Table 2:**
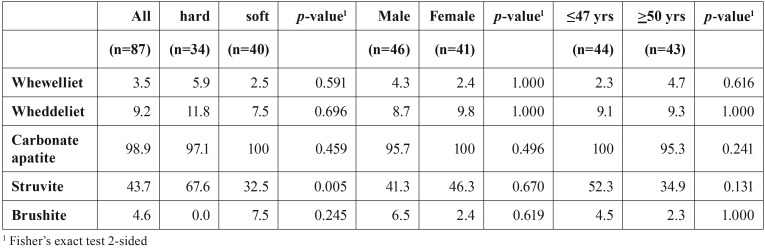
Biochemical analysis of salivary stones, stratified according to hardness of the stone and gender and age of the patient. The number in the table indicate the percentage of the salivary stones that contain the specific component.

Gender of the patient had no significant effects on the biochemical composition of salivary stones. ( Table 1 and  Table 2) 

Stones collected during the second period were subjective classified as ‘hard’ or ‘soft’. Salivary stones classified as hard contained more frequently struvite than stones from the ‘soft’ group (*p*=0.005). ( Table 2)

## Discussion

Insight in the biochemical composition of salivary stones might provide information to clarify the etiopathogenesis of salivary stones, to facilitate diagnosis, to prevent formation and to improve treatment. The present study has shown that the biochemical composition of salivary stones is related to stone-related factors as size and consistency.

Larger stones contain more frequently carbonate ( Table 1). This might be related to the growth of sialoliths, where an initial amorphous core becomes gradually surrounded by concentric laminated layers. These surrounding layers contain carbonate apatite, and differ in degree of mineralization (1). During the growth of the sialolith the number of laminated layers will increase, which might explain the increased contribution of carbonate in larger sialoliths.

The weight of the sialolith is significantly related to the age of the patient (Figure 1). This might relate to age-related changes in circulating serum levels of phosphate. Several studies have reported that the serum phosphate levels are significantly lower in adults above the age of 50 years (13,14). Phosphate acts as crystallization inhibitor (15). Therefore, reduced circulating levels of phosphate could result in less inhibition of crystallization, resulting in larger sialoliths in older individuals. However, this suggestion seems to be contradicted by the biochemical analysis of sialoliths from older individuals. Older individuals showed a trend towards more frequent presence of phosphate in comparison with stones from the ‘young’ group instead of a reduced presence of phosphate ( Table 1). An alternative explanation for the observed association between age and weight of the salivary stone is that in older individuals sialoliths had a longer time to develop.

Brushite was only detected in a relatively small number of salivary stones ( Table 2), much lower than the percentage stones containing carbonate and oxalate. This might be related to the fact that brushite dissolves more rapidly than other calcium minerals like calcium carbonate and calcium oxalate (16).

In the present study, 38% of the salivary stones contained oxalate. Kidney stones are mainly consisting of calcium oxalate, and they occur two to three times more often in men than in women. Watson and co-workers (2010) (17) showed that men with kidney stones have an increased serum total testosterone level, suggesting that this hormone might be related to the deposition of oxalate. To our knowledge, no data are available on the possible relationship between serum total testosterone levels and salivary stones. However, in the present study, salivary stones containing oxalate were more common in men (45.7%) than in women (33.3%), although this difference did not reach statistical significance.

In the present study, protein was detected only in 1% of the salivary stones. This percentage is much lower than previously reported by Sabot and co-workers (6), who identified proteins in more than three quarters of salivary stones. This difference in result could be explained by the fact that the value of infra-red spectrometry in the detection of proteins is rather limited.

The most important limitation of the present study is its retrospective nature, making it dependent on historically collected data. In the eighties of the last century, it was common to use wet chemical techniques for salivary stone analysis because of its low costs. The last 25 years, FT-IR spectrometry for analysis of stones became the analysis of choice. FT-IR spectrometry is a fast and precise technique. The 2013 guidelines on urolithiasis of the European Association of Urology underline the obsolescence of chemical wet analysis and recommend the use of FT-IR for stone analysis (18).

Despite these limitations, the presented historical data suggest that the biochemical composition of salivary stones is related to stone-related factors as size and consistency.
